# Rapid assessment of 3-dimensional intra-tumor heterogeneity through cycling temperature capillary electrophoresis

**DOI:** 10.1186/s13104-023-06437-5

**Published:** 2023-08-11

**Authors:** Anna Połeć, Per Olaf Ekstrøm, Christian Fougner, Therese Sørlie, Jens Henrik Norum

**Affiliations:** 1 Department of Cancer Genetics, Institute for Cancer Research, Radium Hospital, Oslo University Hospital, Oslo, Norway; 2 Department of Tumor Biology, Institute for Cancer Research, Radium Hospital, Oslo University Hospital, Oslo, Norway; 3 Institute for Clinical Medicine, University of Oslo, Oslo, Norway

**Keywords:** Laser capture microdissection, Cycling temperature capillary electrophoresis, Intra-tumor heterogeneity, 3D modeling

## Abstract

**Objective:**

Tumors are heterogeneous three-dimensional masses populated by numerous cell types, including distinct sub-clones of cancerous cells. Various sub-clones within the same tumor mass may respond differently to cancer treatment, and intra-tumor heterogeneity contributes to acquired therapeutic resistance. Thus, one tissue biopsy will in most cases not be representative of the entire genetic landscape of a tumor mass. In this study, we aimed to establish an easily accessible, low cost method to address intra-tumor heterogeneity in three dimensions, for a limited number of DNA alterations.

**Results:**

This study includes analyses of the three-dimensional (3D) distribution of DNA mutations in human colon cancer and mouse mammary gland tumor tissue samples. We used laser capture microdissection for the unbiased collection of tissue in several XY-planes throughout the tumor masses. Cycling temperature capillary electrophoresis was used to determine mutant allele frequency. High-resolution distribution maps of *KRAS* and *Trp53* mutations were generated for each XY-plane in human and mouse tumor samples, respectively. To provide a holistic interpretation of the mutation distribution, we generated interactive 3D heatmaps giving an easily interpretable understanding of the spatial distribution of the analyzed mutations. The method described herein provides an accessible way of describing intra-tumor heterogeneity for a limited number of mutations.

**Supplementary Information:**

The online version contains supplementary material available at 10.1186/s13104-023-06437-5.

## Introduction

Cancers are clonal diseases, which originate from a single cell [1–5]. Tumorigenesis occurs when cells with tumor initiating capacity acquire genomic aberrations with oncogenic potential, e.g. single nucleotide alterations, gene fusions, copy number alterations or small insertions/deletions (indels) [6]. Tumors evolve throughout their lifetime and accumulate genomic aberrations [7, 8] and these additional aberrations may increase or decrease the fitness of a given cell or may have no effect at all. Genomic aberrations that increase the fitness of a tumor cell are often referred to as *drivers*, whereas aberrations with no fitness effect are referred to as *passengers*. Accumulated genomic driver aberrations give rise to various tumor subpopulations, and further clonal evolution is directed by clonal selection (drivers), random genetic drift (passengers), and microenvironmental pressures [9, 10]. Consequently, intra-tumor heterogeneity, in which a tumor contains several distinct sub-clones, each with different genetic characteristics with unique implications for their evolutionary fitness, is likely to arise. Additionally, solid tumors undergo non-tumor infiltration by various cell types, including immune and stromal cells, as well as by blood vessels, which are irregularly distributed throughout the tumor. In summary, the evolution of most tumors results in intra-tumor heterogeneity, which cannot be fully captured by single tissue sample analyses.

Intra-tumor mutation and clonal heterogeneity has been associated with a poor prognosis, and are major causes of acquired therapeutic resistance in cancer [11]. In general, molecular analyses of a single tissue sample from a tumor will usually be unable to provide an exhaustive representation of the tumor’s biology and may consequently fail to identify the attributes driving a patient’s therapeutic response. Moreover, microscopic observations are insufficient to differentiate between mutated and non-mutated cells. Molecular mapping of subclonal architecture in three dimensions is therefore vital to improve our understanding of the total biology of any given tumor. Thus, addressing the spatial distribution of mutations in tumor tissue is important to identify tumor clones that might have different effects on tumor progression and consequently may be of clinical importance.

When studying tumor heterogeneity, there are several important elements that must be considered, such as the region of the tissue that is analyzed and the extent to which this region is representative of the entire tissue volume. Methods of analyzing tumor heterogeneity need to be very sensitive as well as quantitative, because of the tumor composition of diverse cancer cell types and non-mutated cells. Currently, whole genome sequencing and emerging spatial sequencing approaches [12, 13] are capable of addressing tumor heterogeneity, but these assays are extremely costly and require complex laboratory procedures. Cycling temperature capillary electrophoresis (CTCE) is a low cost, targeted method with a mutation detection limit down to 1% [14, 15]. In this study, we used this approach for the spatial modeling of intra-tumor heterogeneity through high-resolution, 3D sampling of tumor tissue for mutational frequency analyses of *KRAS* and *Trp53* in human colon cancer and chemically induced mouse mammary gland tumors, respectively.

## Material and methods

### Tumor samples

Samples used in this study were anonymized specimens of a surgically removed human colon tumor [16, 17] and chemically induced mouse mammary tumors [18]. Under Norwegian legislation, technical and methodological development work that uses anonymized biological material does not require approval by a research ethics committee [14]. Induction and exome sequencing of the chemically induced mouse mammary tumors has been previously described [18].

### Tissue sectioning

Fresh frozen tissue samples were mounted on a cryostat sample holder with a matrix of water-soluble glycols and resins. The sample holder temperature was set to − 20 °C and the knife temperature to − 23 °C. The cryostat was set to cut a series of 12 μm slices, which were subsequently mounted on laser capture microdissection (LCM) membranes or on glass-slides.

### Laser capture microdissection (LCM)

Membrane-mounted 12 μm tissue sections were stained with the morphology marker Giemsa. The stained tissue sections were imaged using a Leica LDM 6000B integrated microscope and LCM system, and repeated patterns of a 8 × 6 grid of 25,000 μm^2^ circles were overlaid on each image. The grids were placed to maximize tissue collection and simultaneously reduce tissue selection bias. It is worth noting that low tissue integrity might influence the density of the data points. The microdissected tissue pieces were collected in 20 μl of 1xThermoPol Reaction Buffer with Proteinase K (0.27 μg/μl). The LCM system was modified to collect samples in strips of PCR tube caps. Following microdissection, the PCR strip caps containing the tissue samples and buffer solution were mounted on a 96-well PCR plate and briefly centrifuged to collect the tissue at the bottom of the wells, and were subsequently incubated at 56 °C for 30 min. Proteinase K was deactivated by raising the incubation temperature to 95 °C for 1 min. After microdissection, the remaining membrane-mounted tissues were digitized using an Olympus VS200 slide scanner.

### Polymerase chain reaction (PCR)

The Primer3 software was used to design human *KRAS* gene PCR primers flanking the DNA target sequences. Subsequent melting point analyses were performed with Variant melting profiles, which is a part of the GSuit HyperBrowser (https://hyperbrowser.uio.no/hb/!mode=advanced). Most *KRAS* gene mutations with clinical impact are in codon 12 and 13 (reviewed in [19]), and due to the high melting temperature of potential PCR products of the mouse *Kras* gene, primers were placed adjacent to codon 12 and 13 in a 42 base pair long fragment. The total length of the product was 84 base pairs including the GC-clamp. Primers were checked for nonspecific amplification by In-Silico PCR (https://genome-euro.ucsc.edu/cgi-bin/hgPcr?hgsid=298560076_UMnEAPrA1vf2p4RE7YXwwPT6G38A). Mutant and wild type melting probabilities were analyzed by WinMelt 2.0 (Bio-Rad), and were used as a guideline for setting the separation temperature in the capillary electrophoresis instrument. The total volume (20 μl) of the collection mixture containing the LCM tissue sample was used as the substrate for PCR amplification and 5 μl of 5 × reaction mixture was then added to each well. The final PCR mixture (with a total volume of 25 μl) consisted of: 1xThermoPol Reaction Buffer with 2 mM MgS04, 0.45 μM forward primer, 0.15 μM reverse primer (with GC-tail), 0.10 μM, fluorescent GC-clamp primer, 500 μM dNTP, 100 μg Bovine Serum Albumin and 0.075 U/μl Taq DNA polymerase. The 96-well plates with the PCR mixtures were sealed with two strips of electrical tape. The PCR temperature cycling conditions were 38 cycles of 94 °C for 30 s, primer specific annealing temperature for 30 s and 72 °C for 60 s. Primer details are listed in Additional file 6: Table S1, and mutation details are listed in Additional file 6: Table S2.

### Cycling temperature capillary electrophoresis (CTCE)

Following DNA amplification by PCR, the mutant allele fraction in each microdissected tissue sample was identified by CTCE, as previously described [14]. The procedure consists of three steps: (1) primer design, including reverse primer with GC-clamp, to specifically amplify the target DNA sequences, (2) PCR-based high-fidelity amplification of the target DNA sequences, and (3) mutant-wild type target sequence separation by CTCE. Steps 1 and 2 are described above. Briefly, in step 3, samples were loaded into the capillaries from 96-well plates by electrokinetic injection at 161 V/cm for 45 s. Electrophoresis was performed with 3% linear acrylamide polymer (with 6 M urea) as separation medium and with a constant field of 145 V/cm. The capillary chamber temperature was cycled 20 times with 3 °C amplitude around the optimal separation temperature for each fragment. This procedure allowed clear identification of double stranded PCR products.

Detection of mutations by CTCE is dependent on an internal standard, which serves as a control of the optimal temperature for separating wild type and mutated target sequences. The internal standard was made by diluting a PCR product from a sample with a high mutant fraction. The diluted (1:1000 in H_2_O) PCR product was used as the template in a re-amplification with a GC-clamp primer containing a fluorophore (ROX) and an unlabeled reverse primer (PCR conditions as described above). In all CTCE runs, the re-amplified PCR product was injected into capillaries prior to sample injection, and thereby used as the internal standard against 6-FAM labeled potential mutation-containing PCR products. Wild type, mutated and heteroduplex products were detected as individual peaks in the electropherograms [15]. The mutant fraction for each sample was calculated by dividing the area under the mutant peak plus half the area of the two heteroduplex peaks by the total area of all peaks (Additional file 5: Fig. S1).

### Data analyzes and visualization

To determine if the calculated *KRAS* mutation distributions in the human colon cancer sample were significantly different from 0, confidence intervals for each mutant frequency ($\widehat{p}$) were calculated. First we checked whether the proportions could be assumed to follow normal distribution by checking that *n* × $\widehat{p}\ge$ 5 and *n* × (1 −  $\widehat{p}$)$\ge$ 5, where *n* is the sum of all areas under the curves (AUCs) in each electropherograms (the denominator in the calculation of the proportion). All values were $\ge$ 5, except two cases that showed only wild type signal. Thus, we can assume normal distribution. Next, we constructed the standard 95% confidence interval by calculating the margin error for the confidence interval (*z*) using the norm.s.inv function in Microsoft Excel, and subsequently calculating the lower ($\widehat{p}-z \sqrt{\frac{\widehat{p} x (1-\widehat{p})}{n}}$) and upper limits $(\widehat{p}+z \sqrt{\frac{\widehat{p} x (1-\widehat{p})}{n}}$). The confidence intervals were narrow, and no confidence intervals included the value 0 (Additional file 1). Thus, it is reasonable to assume that the calculated mutant fractions in each LCM tissue piece represents the allele fraction of the addressed mutations. These results confirmed the robustness of the CTCE method.

Data was analyzed by creating two-dimensional (2D) heatmaps using the Plotly package [20] for R. Missing values were imputed using linear interpolation in the Akima package [21]. Interactive 3D heatmaps were created in Matlab using meshgrid (x, y, z), which returns 3D grid coordinates defined by the vectors x, y, and z (Additional file 2). The sampling grid represented by X and Y, is the grid of the laser captured microdissected tissue pieces while Z represents tissue sections from different planes of the tumor mass. Interpolation using natural neighbor interpolation with continuity C^1^ (except at sample points) on the 3D data set was performed with scatteredInterpolant, which uses a Delaunay triangulation of the scattered sample points [22]. Isosurfaces as 3D surface representations of points with equal values in a 3D data distribution were created as representations of equal mutant fractions in the 3D data distribution.

### Data availability

The mouse mammary exome sequence data used in the current study is available through the European Nucleotide Archive, accession number PRJEB29718. The PCR data used to generate the heatmaps presented herein are available in Additional files 3 and 4.

## Results

**Fig. 1:**
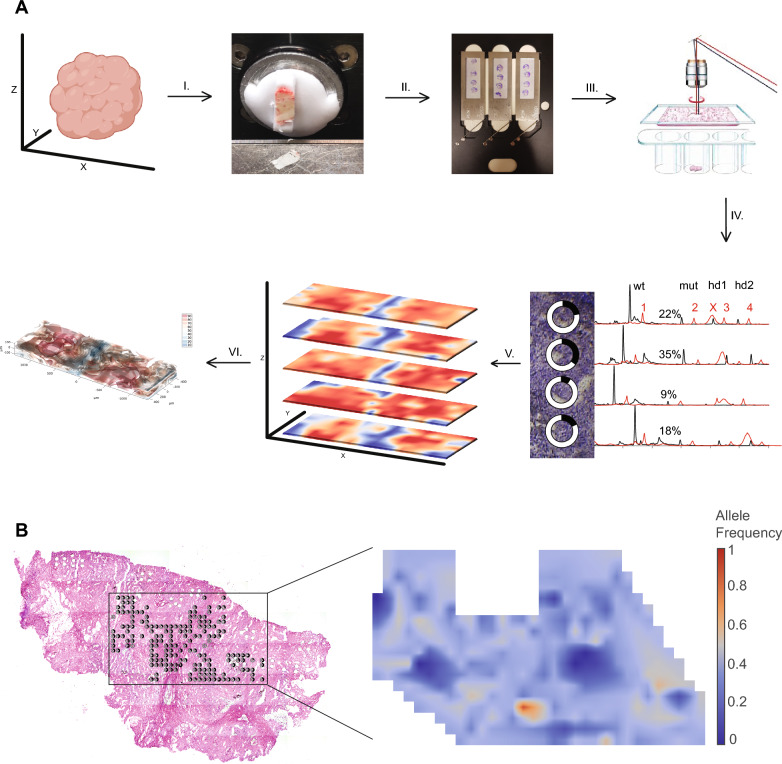
Workflow for assessment and visualization of three-dimensional (3D) DNA point mutation distribution in tumor tissues, and distribution of *KRAS* mutation in XY-plane. **A** Tumor tissue was dissected free from surrounding tissue, flash frozen and mounted for cryosectioning (I). Sectioned tissue was applied to LCM membranes, fixed and stained with Giemsa (II). Laser capture microdissection was performed, and tissue areas of interest captured (III). PCR products containing specific mutations were detected by cycling temperature capillary electrophoresis* (IV). The frequency distribution of each mutation (in %) was visualized with heatmaps for each analyzed tissue section level (V). 3D mutation frequency and distribution models were generated to give a holistic overview of the heterogeneous tumor tissue (VI). **B** *KRAS* mutations in colon cancer tissue were mapped using laser capture microdissection and CTCE, and are represented by circles. The extent to which the circle rim is filled represents the *KRAS* mutation allele frequency in each LCM tissue sample. The allele frequencies were translated to a heatmap for easier interpretation of the distribution pattern. *Red curve: internal standard; 1: wild type; 2–4 heteroduplexes; X: single strand: Black curve: sample curve; *wt* wild type, *mut* mutant; *hd* heteroduplex

We have established a simple, low cost procedure to address the heterogeneous distribution and the frequency of each mutation (calculated for every microdissected area) in tumor tissue using laser capture microdissection of cryopreserved tissue and PCR with subsequent CTCE. Tumor heterogeneity does not only vary in the XY plane but also along the Z axis of the three-dimensional tumor. Thus, to expand the view of tumor heterogeneity to three dimensions, we generated 3D heatmaps visualizing mutation frequencies in the XYZ space. The workflow is outlined in Fig. 1A.

Initially, we established our method to determine the heterogeneous distribution of mutated *KRAS* in the XY-plane in an anonymized human colon cancer tumor tissue sample. To reduce potential selection bias, the LCM samples were collected using fixed 8 × 6 grids. Tissue was microdissected from 8 adjacent 8 × 6 grids overlaying the tissue section (Fig. 1B). Overall, 201 of the 384 microdissected tissue pieces were lost or contained insufficient cells for DNA extraction. The frequency of mutated *KRAS* in every piece of microdissected tissue was calculated and represented visually as circles with the rims filled to different degrees (Fig. 1B). Based on these frequency calculations, a heatmap was generated reflecting the distribution of the *KRAS* mutation across the 2D tumor tissue surface (Fig. 1B).

**Fig. 2:**
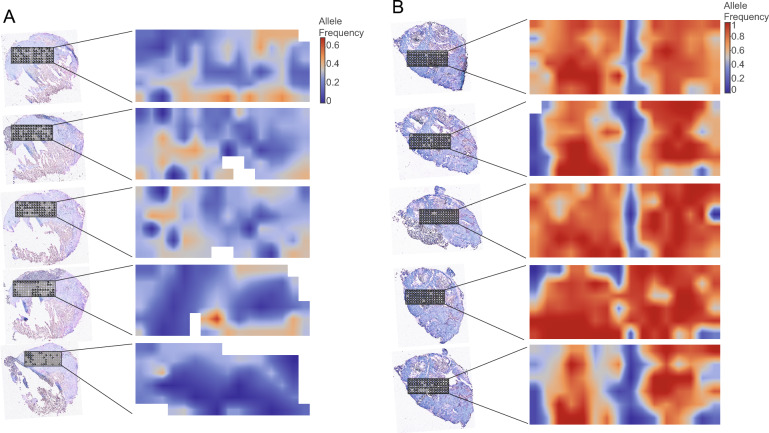
Distribution of *Trp53* and *Kras* mutations in mouse mammary gland tumor tissue. Tissue areas from five tissue sections separated in the Z-plane were laser capture microdissected and mutation frequencies mapped with circles. The extent to which the circle rim is filled represents the *Trp53* (**A**) and *Kras* (**B**) mutation allele frequency in each LCM tissue sample. LCM areas that did not generate data are marked with filled grey circles. The allele frequencies were translated to a heatmap for each Z-plane tissue section for easier interpretation of distribution patterns

Next, we validated this procedure by generating 2D heatmaps of mutated *Trp53* (Fig. 2A) and mutated *Kras* (Fig. 2B) previously identified by DNA sequencing in a study of chemically induced mouse mammary gland tumors [18]. Mutated *KRAS* is a well-known oncogene (reviewed in [19]) while the tumor suppressor *TP53* gene (*Trp53* mouse analogue) is the most frequently mutated gene in human cancers (reviewed in [23]). Mapping the spatial distribution patterns of mutations in these two genes is of importance to increase our understanding of mutated KRAS and TP53 biology, and their role in tumor development.

The mouse mammary gland tumor tissue was collected from tumor sections spanning 400 μm and 200 μm along the Z-axis, respectively. Of the 96 potential data points from each of the five Z-plane tissue sections analyzed for the *Kras* mutation, 1, 6, 2, 3 and 9 microdissected areas did not generate data due either to lost tissue, absent tissue at the collection point, or an insufficient number of cells. More of the potential data points were lost from the five Z-plane tissue sections used for *Trp53* mutation analysis: 24, 20, 29, 55 and 67, respectively. The mutation frequencies and missing data are indicated in Fig. 2. To visualize the distribution of the mutations in each XY-plane, 2D heatmaps were generated (Fig. 2).

**Fig. 3:**
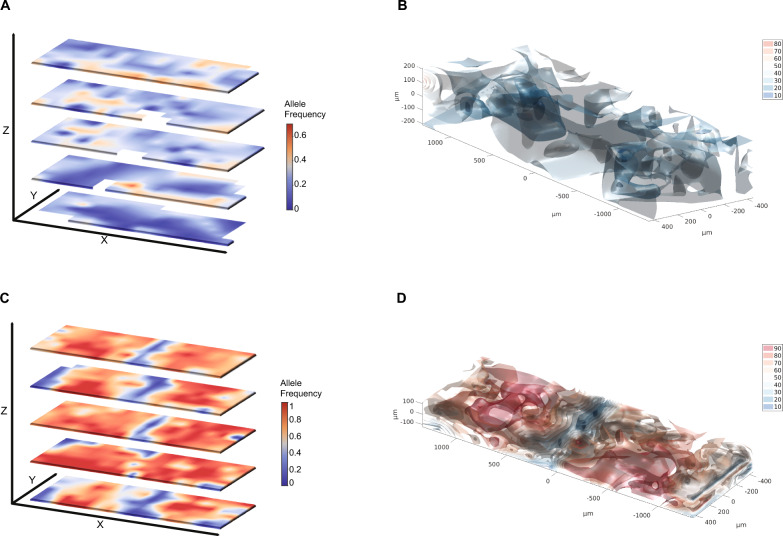
2D and 3D heatmaps of allele frequency and distribution in tumor tissue. 2D heatmaps of *Trp53* (**A**) and *Kras* (**C**) mutant allele frequencies in independent z-planes of mouse mammary gland tumors. 3D heatmaps of the same *Trp53* (**B**) and *Kras* (**D**) mutant allele frequencies were generated from the independent z-planes of mouse mammary gland tumors. Interactive 3D heatmaps are available in Additional file 5: Figs. S2 and S3

Finally, we generated 3D heatmaps representing the distribution of the *Trp53* (Fig. 3A and B) and *Kras* (Fig. 3C and D) mutations. These can be viewed as interactive heatmaps (Additional file 5: Figs S2 and S3) to give a holistic and easy-to-understand overview of the spatial distribution of gene mutations, clearly illustrating intra-tumor heterogeneity.

## Limitations

Our method is a useful tool to address and present intra-tumor heterogeneity in an easy interpretable 3D format. However, there are possibilities to improve it further. For example, a microscope with higher resolution connected to the LCM apparatus would enable pathological examination of the microdissected areas and provide further clinically relevant information.

The major limitation to our work is the same issue as the one we are trying to address: intra-tumor heterogeneity. When frozen tumor tissue is sectioned and placed on LCM membranes for microdissection, it is possible that the tissue sections may not be perfectly aligned, thus introducing a “twist” in the x–y plane when sampling tissue along the z-axis. However, we sampled several points in each plane and our goal is not to follow a specific coordinate through several z planes, rather we aim to describe the total level of heterogeneity in each plane, and subsequently throughout the tumor volume. To directly compare specific x–y coordinates through several z planes, two physical guiding points might be introduced throughout the entire tumor volume, e.g. by piercing two holes through the tumor tissue prior to sectioning.

## Conclusion

In this methodological study, we have applied focused laser capture microdissection and cycling temperature capillary electrophoresis to measure mutant fractions in genomic DNA with very low amounts of input DNA. The mutation detection limit is about 1%, and the entire laboratory procedure takes approximately six hours. The method allows the unbiased collection of tumor tissue, and visualization of spatial intra-tumor heterogeneity at relative low cost. Our method combines several previously well-established laboratory techniques, and statistical calculations to generate 3D interactive heatmaps. Moreover, 3D mapping of intra-tumor heterogeneity provides an additional level of understanding of the complex tumor biology involved in tumor evolution.

### Supplementary Information

**Additional file 1: **Normal distribution and standard 95% confidence interval calculations.

**Additional file 2: **MATLAB script used generate mouse Trp53 and Kras 3D heatmaps.

**Additional file 3: **R script and data used to generate human KRAS 2D heatmap.

**Additional file 4: **R script and data used to generate mouse Trp53 and Kras 2D heatmaps.

**Additional file 5: Figure S1.** CTCE electropherograms illustrating 20% mutation fraction. The mutant fraction is calculated to 20%, when the area under the wild-type, mutant and heteroduplex1 and thereroduplex2 are 10000, 1000, 2000, and 2000, respectively. **Figure S2.** Interactive 3D heatmaps of *Trp53* mutation distribution. **Figure S3.** Interactive 3D heatmaps of *Kras* mutation distribution.

**Additional file 6: Table S1.** Primers used for PCR. The reverse primers were 1/2 CG-clamp tailed (in bold). **Table S2.** *KRAS*, *Kras* and *Trp53* mutation information.

## Data Availability

The mouse mammary exome sequence data used in the current study is available through the European Nucleotide Archive, accession number PRJEB29718. The mutant allele frequency data is available in Additional files 1, 3 and 4.

## Abbreviations

CTCE: Cycling temperature capillary electrophoresis
LCM: Laser capture microdissection
