# Supplementary material for: Rapid assessment of 3-dimensional intra-tumor heterogeneity through cycling temperature capillary electrophoresis
Source: BMC Res Notes. 2023 Aug 11;16:167. doi: 10.1186/s13104-023-06437-5 (PMC10416412; doi:10.1186/s13104-023-06437-5)
Supplement: Supplementary file 5 — Additional file 5: Figure S1. CTCE electropherograms illustrating 20% mutation fraction. The mutant fraction is calculated to 20%, when the area under the wild-type, mutant and heteroduplex1 and thereroduplex2 are 10000, 1000, 2000, and 2000, respectively. Figure S2. Interactive 3D heatmaps of Trp53 mutation distribution. Figure S3. Interactive 3D heatmaps of Kras mutation distribution. [file 13104_2023_6437_MOESM5_ESM.zip › Additional file 5/Supplementary_figure_2_TP53_v2.xhtml]

Matlab X3D


# *Trp53* mutation distribution in 3D. Left mouse button to rotate, Ctrl left to move, wheel to zoom. Different colors represent different allele fractions.

 
