# Supplementary material for: Rapid assessment of 3-dimensional intra-tumor heterogeneity through cycling temperature capillary electrophoresis
Source: BMC Res Notes. 2023 Aug 11;16:167. doi: 10.1186/s13104-023-06437-5 (PMC10416412; doi:10.1186/s13104-023-06437-5)
Supplement: Supplementary file 6 — Additional file 6: Table S1. Primers used for PCR. The reverse primers were 1/2 CG-clamp tailed (in bold). Table S2. KRAS, Kras and Trp53 mutation information. [file 13104_2023_6437_MOESM6_ESM.docx]

**Table S1**: Primers used for PCR. The reverse primers were 1/2 CG-clamp tailed (in **bold)**.

| Name | Label | Strand orientation 5' - 3' | Annealing temperature | Separation temperature |
| --- | --- | --- | --- | --- |
| KRAS-Forward-human | - | CATTATTTTTATTATAAGG | 47.5°C | (54°C-51°C)x20 |
| KRAS-Revers-human | - | **CCCGCCGCCCCCGCCCGGG**TCTATTGTTGGATCATATTC |  |  |
| Kras-Forward-mouse | - | TATAAACTTGTGGTGGTTGGAGCT | 60°C | (61°C-58°C)x20 |
| Kras-Revers-mouse | - | **CCCGCCGCCCCCGCCCGGG**TCGTCAAGGCGCTCTTGCCTAC |  |  |
| Trp53-Forward-mouse | - | AAAGTCTGCCTGTCTTCCAGATACT | 57°C | (56°C-53°C)x20 |
| Trp53-Reverse-mouse | - | **CCCGCCGCCCCCGCCCGGG**TCCGGGTGGAAGGAAATTTGTATCCC |  |  |
| GC-clamp* | FAM | CGCCCGCCGCGCCCCGCGCCCGTCCCGCCGCCCCCGCCCGGG |  |  |
| GC-clamp | ROX | CGCCCGCCGCGCCCCGCGCCCGTCCCGCCGCCCCCGCCCGGG |  |  |

* Ekstrøm PO, Nakken S, Johansen M, Hovig E. Automated amplicon design suitable for analysis of DNA variants by melting techniques. BMC Res Notes. 2015;8:667.

**Table S2**: *KRAS*, *Kras* and *Trp53* mutation information

| Sample | Specie | Gene | AA change | AA change symbol | Mutation coordinate | Reference nucleotide | Alternative nucleotide |
| --- | --- | --- | --- | --- | --- | --- | --- |
| Colon cancer | Human | *KRAS* | - | - | Assumed codon 12 or 13 | - | - |
| S176_14_2* | Mouse | *Kras* | p.Gly13Arg | p.G13R | chr6:145246769 | C | G |
| S160_14_2* | Mouse | *Trp53* | p.Glu201* | p.E201* | chr11:69588676 | G | T |

*Fougner C, Bergholtz H, Kuiper R, Norum JH, Sorlie T. Claudin-low-like mouse mammary tumors show distinct transcriptomic patterns uncoupled from genomic drivers. Breast Cancer Res. 2019;21:85.
